# Biomimetic Mineralizing Agents Recover the Micro Tensile Bond Strength of Demineralized Dentin

**DOI:** 10.3390/ma11091733

**Published:** 2018-09-14

**Authors:** Luiz Filipe Barbosa-Martins, Jossaria Pereira de Sousa, Lívia Araújo Alves, Robert Philip Wynn Davies, Regina Maria Puppin-Rontanti

**Affiliations:** 1 Department of Pediatric Dentistry, Piracicaba Dental School, State University of Campinas, Piracicaba 13414-903; Brazil; flpmarttins@gmail.com (L.F.B.-M.); jossariasousa@gmail.com (J.P.d.S.); 2 Department of Oral Diagnosis, Piracicaba Dental School, State University of Campinas, Piracicaba 13414-903, Brazil; liviaaalves@hotmail.com; 3 Division of Oral Biology, School of Dentistry, Faculty of Medicine & Health, University of Leeds, Leeds LS9 7TF, UK; R.P.W.Davies@leeds.ac.uk; 4 Departments of Pediatric Dentistry and Restorative Dentistry, Piracicaba Dental School, University of Campinas, Piracicaba 13414-903, Brazil

**Keywords:** dentin, desmineralization, microtensile bond strength

## Abstract

Biomimetic remineralization is an approach that mimics natural biomineralization, and improves adhesive procedures. The aim of this paper was to investigate the influence of Dentin Caries-like Lesions (DCLL)-Producing Model on microtensile bond strength (μTBS) of etch and rinse adhesive systems and investigate the effect of remineralizing agents such as Sodium Fluoride (NaF), MI Paste™ (MP) and Curodont™ Repair (CR) on caries-affected dentin (n = 6). Nine groups were established: (1) Sound dentin; (2) Demineralized dentin/Chemical DCLL: (3) Demineralized dentin/Biological DCLL; (4) Chemical/DCLL + NaF; (5) Chemical/DCLL + MP; (6) Chemical/DCLL + CR; (7) Biological/DCLL + NaF; (8) Biological/DCLL + MP; (9) Biological/DCLL + CR. Then all dentin blocks were subjected to a bonding procedure with Adper™ Single Bond 2 adhesive system/Filtek Z350XT 4 mm high block, following this they were immersed in deionized water/24 h and then sectioned with ≅1 mm^2^ beams. The μTBS test was conducted at 1 mm/min/500 N loading. Failure sites were evaluated by SEM (scanning electron microscopy (150×). μTBS data were submitted to factorial ANOVA and Tukey’s test (*p* < 0.05). The highest values were found when demineralized dentin was treated with MP and CR, regardless caries lesion depth (*p* < 0.05). There was a predominance of adhesive/mixed in the present study. It was concluded that the use of the artificial dentin caries production models produces differences in the μTBS. Additionally MP and CR remineralizing agents could enhance adhesive procedures even at different models of caries lesion.

## 1. Introduction

During the execution of routine dental restorations, the hybrid structure formed by the dental bonding procedure occurs through the interaction and subsequent polymerization of monomers around the demineralized collagen matrix [1]. The oral cavity is a severe environment for the resin-dental bond to survive for a reasonable length of time, with thermomechanical changes, chemical attacks by acids and enzymes and other factors posing routine daily challenges. Therefore, to achieve effective and stable bonding, the preservation of dentin collagen is critical, since collagen represents the major organic component of the dentin matrix [2].

Caries is among the most common diseases worldwide [3], and the immediate bond strengths to caries-affected dentin are commonly 20–50% lower than to sound dentin [4,5,6]. The restoration of the normal conditions of the mineral content of the caries-affected dentin, prevents the action of enzymes in addition to providing an increased bond durability [7].

Biomimetic remineralization mimics the process of natural biomineralization by replacing demineralized collagen matrix water with apatite crystallites [7]. Caries-affected dentin is comprised of about 14–53% of water compared with sound dentin, which exhibits a much lower value [8]. Therefore, by replacing water with minerals at the dentin–resin interface, this would increase the mechanical properties and inhibit water-related hydrolysis [9].

It has recently been demonstrated that the use of remineralizing agents in dentin could recover the mechanical properties of the substrate [10]. In addition to sodium fluoride (NaF) and sodium phosphate (Na_3_PO_4_), casein phosphopeptide amorphous calcium phosphate (CPP-ACP), which is derived from milk protein, can release calcium phosphate assisting in enamel and dentin remineralization [11]. It acts mainly by inhibiting demineralization and enzymatic degradation [12]. Furthermore, recent studies have shown that the use CPP-ACP has no negative effect on bond strength [13,14]. The peptidic biomimetic matrix ‘P_11_-4’, which has been incorporated into a clinical product (Curodont™ Repair) has shown encouraging results in early clinical trials. It has been shown to improve the visual appearance of carious lesions and increases the opacity on X-rays after treatment of proximal caries [15,16]. Additionally, following the application of P_11_-4 and subsequent bonding procedures an increased resin-dentin bond strength has been observed [17].

The restructured demineralized collagen matrix found in caries-affected dentin process by interventions such as Sodium Fluoride (NaF), CPP-ACP contained in MI Paste™ (GC International) and P_11_-4 peptide contained in Curodont™ Repair (Credentis AG), prior to adhesive procedures by an etch-and-rinse adhesive system (Adper™ Single Bond 2 (3M ESPE) in the demineralized dentin could be a promising proposal for adhesive clinical procedures.

Clinical binding procedures simulated by mechanical methods (i.e., TMBS, μTBS) often use artificial demineralized dentin. However, the lack of standardization of caries lesions creates technical difficulties for evaluation [18]. In vitro models have been used to produce demineralized dentin under controlled conditions [19,20,21,22]. Chemical methods provide superficial dentin demineralization, resulting in a substrate with similar hardness compared to natural caries-affected dentin [19]. Conversely, the microbiological method promotes an excessive softening of dentin, but with a more comparable morphological pattern of collagen degradation to natural caries lesions [19,20,21,22]. Pacheco et al., 2013 [23], evaluating molecular and structural lesions related to dental caries, produced by the chemical (GC), biological (GB), in situ (GIS) and natural (CNG) approaches, showed similar and lower surface hardness between CNG and GB of which GC and GIS, lower mineral content (Ca^2+^ and PO_4_^3−^) for GB and GNC than GC and GIS. Therefore, the structure and mechanical properties are different with respect to the caries model production and the remineralizing agents may act differently in the adhesion procedures depending on the model of caries lesions used.

To ascertain the potential efficacy of the bonding procedure carried out on remineralized dentin we aimed to evaluate different remineralization treatments and the method used in producing the simulated dentin-like caries lesions on the micro tensile bond strength of remineralized dentin. The hypothesis was that the Dentin Caries-like Lesions Producing Model and the remineralizing agents (Sodium Fluoride-(NaF), MI Paste™-(MP) and Curodont™ Repair-(CR)) affect microtensile bond strength-μTBS of etch-and-rinse adhesive system on caries-affected dentin.

## 2. Materials and Methods

**Figure 1:**
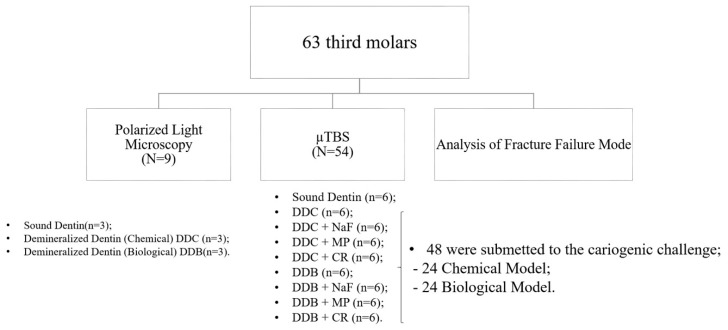
Experimental design. DDC—demineralized dentin provided by chemical model; DDC/NaF-DDC + 2% NaF; DDC/MP-DDC + MI Paste™; DDC/CR-DDC + Curodont™ Repair; DDB—demineralized dentin provided by biological model; DDB/NaF-DDB + 2% NaF; DDB/MP-DDB + MI Paste™; DDB/CR-DDB + Curodont™ Repair.

**Figure 2:**
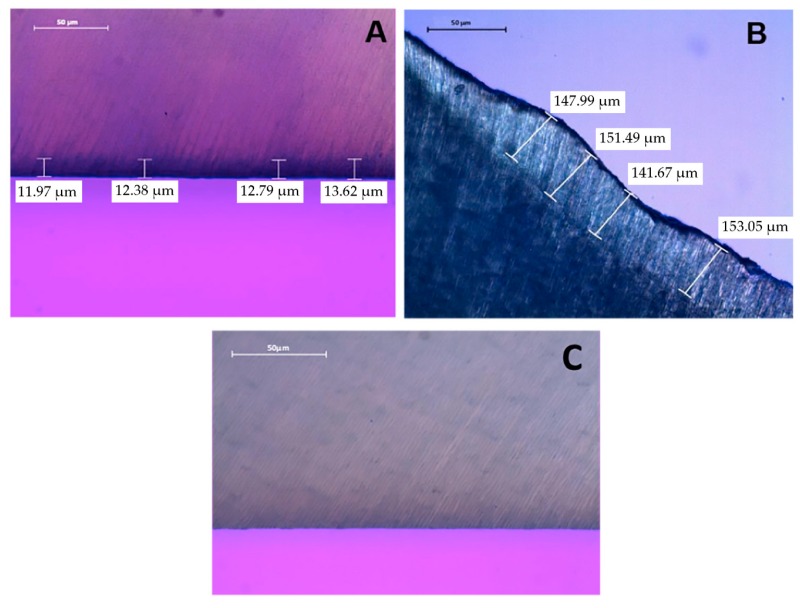
(**A**) Artificial caries lesions provided by Chemical Model; (**B**) Artificial caries lesions provided by Biological Model after removing the softened tissue; (**C**) Sound Dentin.

Sixty-three sound human third molars were collected with patients’ informed consent, as approved by the Ethics Committee of Piracicaba Dental School, University of Campinas (Protocol number 37634814.5.0000.5418). The teeth were stored in 0.1% thymol solution at 4 °C for no longer than 2 months after extraction. A 4.0 mm coronal dentin slice from each tooth was obtained by sectioning 2.0 mm below cement-enamel junction (CEJ), and 2.0 mm above CEJ, using a slow-speed water-cooled diamond saw (Isomet 1000, Buehler Ltd., Lake Bluff, IL, USA). Six dentin slices were used for sound dentin (control group-CG), and the others were randomly assigned into 2 groups (n = 24), according to the caries method production. The dentin surface of each specimen was wet polished with a 600-grit SiC paper (Arotec, São Paulo, Brazil) for 30 s to create a standardized smear layer. The dentin surfaces were carefully examined under a stereomicroscope at ×50 magnification to confirm the absence of enamel islets. The specimens were immediately subjected to production of caries in vitro. The group distribution is illustrated in Figure 1. To check the caries dentin depth, three teeth were chosen from each group and probed using a polarized light microscope (Figure 2).

### 2.1. Artificial Dentin Caries-Like Lesions (DCLL) Production Protocols

Sixty dentin slices (54 teeth for µTBS and 6 for polarized light microscopy) were randomly assigned into 2 groups according to dentin-like caries lesions producing models: chemical (carboxymethylcellulose acid gel) and biological (Streptococcus mutans—UA159 biofilm).

#### 2.1.1. Chemical Model

The specimens were submerged in vials containing 5 mL of 6% carboxymethylcellulose acid gel (0.1 M lactic acid titrated to pH 5.0 in a KOH solution) at pH 5.0 and 37 °C. The specimens remained in the gel for 48 h without renewal [23]. This model has been reported to supposedly provide a demineralized dentin similar to that of caries affected dentin.

#### 2.1.2. Biological Model

The specimens were fixed with orthodontic wire on the lids of glass vials containing 250 mL of sterile deionized water and were sterilized with gamma radiation (14.5 kGy dose) for 60 h (Pacheco et al., 2013). Then, they were transferred to another glass vial containing 250 mL of sterile brain-heart infusion (BHI) broth (LabCenter, São Paulo, Brazil) supplemented with 0.5% yeast extract (LabCenter, São Paulo, Brazil), 0.5% glucose (LabCenter, São Paulo, Brazil), 1% sucrose (LabCenter, São Paulo, Brazil) and 2% *S. mutans* (UA159, ATCC, Oklahoma, OK, USA) incubated at 37 °C and supplemented with 10% CO_2_, pH of around 4.0. Starter culture was transferred into 250 mL of fresh BHI and grown for 4 h at 37 °C under aerobic conditions. Optical density at 550 nm (A550) of all bacterial suspensions was adjusted to 0.05 prior to inoculation. Inoculation occurred only in the first day of the experiment, but the broth was renewed every 48 h over a 7-day period. The broth was Gram stained daily to monitor contamination. The resulting biofilm formed over the teeth was removed with gauze and the softer dentin layer was removed using #6 carbide drills; the removal ceased when dentin appearance was like that of caries-affected dentin [23].

### 2.2. Polarized Light Microscopy (PLM)

After providing dentin caries-like lesions, three specimens of each dentin caries-like lesions-producing models and control group were sectioned perpendicular to the occlusal surface to obtain slices. The two more central slices of each tooth were selected and polished with #800, #1200, #2400 and #4000-grit silicon carbide (SiC) paper (Buehler, Lake Buff, IL, USA), obtaining a 0.15 µm dentin thickness. Dentin slices were analyzed for the depth of demineralization on PLM (Leica DMLP, Leica microsystems, Wetzlar, Germany). The dentin slices were kept in 100% humidity throughout the investigation. Depth caries lesions were measured in a PLM (Leica DMLP, Leica microsystems) using 20×/0.4 (corr.) objective. Standard settings for contrast, brightness and light were used for all images. Four measurements were made in different parts of the same lesion from the lesion border to the deepest part of the lesion, for each dentin caries-like lesion model. An average depth for each specimen was calculated from the individual values based on depth difference between dentin caries-like lesions and sound dentin on the same specimen. Dentin caries-like lesions observed in the specimens subjected to Chemical Model presented x = 12.69 µm average depth and Biological Model x = 148.55 µm, measured using PLM.

### 2.3. Dentin Surface Treatment

Thirty-six teeth were assigned to 6 groups according to the remineralization treatment: demineralized dentin by chemical model (DDC) + treated with 0.2% NaF Solution (1 min)—DDC/NaF; DDC + treated with MI Paste™ (1 min)—DDC/MP; and DDC + treated with Curodont™ Repair—DDC/CR applied (5 min) plus Ca^2+^ and PO_4_^3−^ Solution (1 min); demineralized dentin by biological model (DDB) + 0.2% NaF Solution—DDB/NaF; DDB + treated with MI Paste™ applied (1 min)—DDB/MP; and DDB + treated with Curodont™ Repair—DDB/CR applied (5 min) plus Ca^2+^ and PO_4_^3−^ Solution (1 min).

The specimens of groups DDC/NaF and DDB/NaF were remineralized using 0.1 mL of the 0.2% NaF. 0.2% NaF solution was applied on demineralized dentin surface and left dry for 1 min at room temperature for the groups DDC/NaF and DDB/NaF. 0.1 mL of the MI Paste™ was applied onto the surface of the specimens from DDC/MP and DDB/MP groups with microbrush for 1 min at room temperature, the excess paste was removed by washing with deionized water. For DDC/CR and DDB/CR groups, 50 µL of the Curodont™ Repair was applied and left for 5 min, then, a Ca^2+^ and PO_4_^3−^ solution was applied and left onto surface for 1 min. For all treatments, the solution excess was removed with absorbent paper.

### 2.4. Bonding Procedures

A single operator applied the adhesive according to the manufacturer’s instruction (Table 1). An LED light-curing unit (Bluephase, Ivoclar Vivadent; Schaan, Liechtenstein) was set to the low power mode with a light intensity of 650 mW/cm^2^. A nanohybrid resin composite (Filtek Z350 XT, A2 (3M ESPE, St. Paul, MN, USA)) was used to create resin composite buildups in four layers of 1 mm each [17]. Each layer was light cured for 20 s, followed by a final polymerization of 60 s. The specimens were then stored at 100% humidity at 37 °C for 24 h.

### 2.5. Microtensile Bond Strength Test (µTBS)

After storage, the specimens were sectioned perpendicularly to the resin/dentin interface to produce dentin–resin beams with 1 mm^2^ at cross-sectional area, using a low speed diamond saw (ISOMET 1000, Buehler Ltd., Lake Buff, IL, USA). From six to eight beams were obtained per tooth, each beam was measured with a digital caliper (Mitutoyo; Kawasaki, Japan) to determine the cross-sectional area. All beams were kept in deionized water for 24 h.

For µTBS measurements, each beam was fixed to a microtensile device with cyanocrylate glue (Super Bonder (#1883519), Loctite, Henkel Corp., Rocky Hill, CT, USA), and tested in a universal testing machine (DL 2000, EMIC, Equipment and Systems Ltda., São José dos Pinhais, Brazil). The test was carried out with a 500 N load at 1.0 mm/min cross speed until failure. The µTBS values were expressed in MPa.

### 2.6. Analysis of Failure Mode

All the fractured specimens from the microtensile bond strength analysis were assessed to determine the failure mode using SEM at ×50 and ×150 magnifications. The fractured surfaces of the beams were paired, air dried, mounted on aluminum stubs, gold coated, and examined by SEM (JSM-5600LV, JEOL; Tokyo, Japan), operated at 15 kV. The failure patterns were classified according to the following categories: adhesive, mixed (involving resin composite, adhesive and/ or dentin), cohesive failure in the resin composite, and cohesive failure in dentin [17,25,26].

### 2.7. Statistical Analysis

Bond strength values for each group were analyzed by Shapiro–Wilk test (R Software version 3.4.3, The R Foundation for Statistical Computing, Vienna, Austria) in order to assess the normality of the data distribution. Factorial ANOVA and post hoc Tukey test (R Software version 3.4.3, The R Foundation for Statistical Computing, Vienna, Austria) were used to determine statistically significant differences between factors: the dentin-like caries lesions model (two levels—chemical and biological models) and dentin remineralization treatment (four levels—SD, DD, NaF + DD, CPP-ACP + DD and P_11_-4 + DD) on dentin/resin bond strength, and additional Dunnett test to determine statistically significant differences between the experimental groups and the control group (sound dentin). The Kruskal Wallis test was used to evaluate the failure mode. The R Software version 3.4.3 (The R Foundation for Statistical Computing, Vienna, Austria), was used to perform the tests. Statistical difference was set at α = 5%.

## 3. Results

The images of the lesions observed in Chemical Model x = 12.69 (average depth) (A) and Biological Model x = 148.55 (B) using polarized light microscopy are displayed in Figure 2. 

Factorial ANOVA revealed a significant interaction between studied factors: dentin caries-like lesion model and remineralization treatment (*p* < 0.001). In addition, there was a statistically significant difference concerning artificial dentin caries-like lesion model (*p* < 0.001), and also between treatments (*p* < 0.001).

As shown in Table 2, a Tukey test (R Software version 3.4.3, The R Foundation for Statistical Computing, Vienna, Austria) revealed that µTBS values of NaF to demineralized dentin for both, chemical and biological dentin caries-like lesion models, were significantly lower than other remineralizing agents (*p* < 0.001). Biological DCLL model significantly reduced the microtensile bond strength when the dentin was treated by NaF and Curodont™ Repair (*p* < 0.001). Chemical DCLL model provided higher μTBS than the biological one, when demineralized dentin was treated by NaF and Curodont Repair (*p* < 0.05). In addition, dentin demineralized by the chemical DCLL treated with Curodont™ Repair provided the highest bond strength, and there was no significant difference from MI Paste™ for the same dentin condition, and they were significantly higher than sound dentin (*p* < 0.05). However, when DCLL was treated with MI Paste™, there was no influence regardless of the DCLL model (*p* > 0.05) and they were significantly higher than sound dentin (*p* < 0.05). However, only when NaF was used there was an observed lower μTBS than sound dentin (*p* < 0.05). For both DCLL models, demineralized dentin treated with NaF, MI Paste™ and Curodont™ Repair showed significant higher μTBS than demineralized dentin (*p* < 0.01). Demineralized dentin group showed the lowest bond strength for all groups (*p* < 0.01) (Table 2).

**Figure 3:**
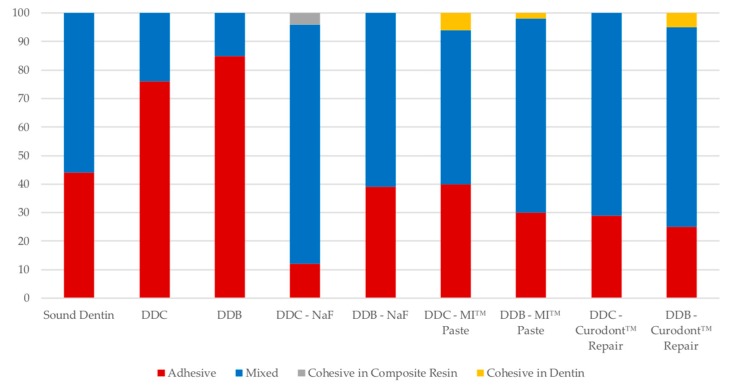
Distribution of failure modes. DDC—Demineralized Dentin by chemical model; DDB—Demineralized Dentin by biological model; NaF—Sodium Floride; MI Paste™-CPP-ACP—Casein phospopeptide-amorphous calcium phosphate; Curodont™ Repair—P_11_-4—Peptide self-assembly.

The failure modes of specimens are shown in Figure 3. The failure modes of DDC (76%) and DDB (85%) specimens were predominantly adhesive failure; Mixed failure were found for DDC-NaF (84%), DDB-NaF (61%), DDC-MI Paste™ (54%), DDB-MI Paste™ (68%), DDC-Curodont™ Repair (71%) and DDB-Curodont™ Repair (70%); cohesive failure in composite resin was observed in the DDB-NaF (4%); cohesive failure in dentin was observed in the DDC-MI Paste™ (6%) DDB-MI Paste™ (2%) and DDB-Curodont™ Repair (5%) groups. The failure patterns were often adhesive and mixed for all groups. There was no statistically significant difference between the fracture type by Kruskal-Wallis’s test, concerning DCLL model (*p* = 0.9967).

**Figure 4:**
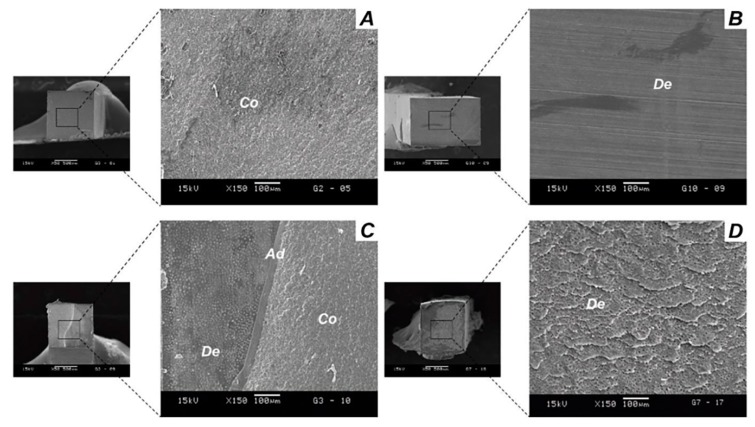
SEM image of failure modes. (**A**) Cohesive failure in Resin Composite; (**B**) Adhesive failure; (**C**) Mixed failure and (**D**) Cohesive failure in Dentin. Abbreviations shows areas of **Co**. Composite; **Ad**. Adhesive; **De**. Dentin.

Figure 4 shows representative SEMs for the fracture patterns observed for the different groups. Figure 3A—Cohesive failure on composite; 3B—Adhesive failure; 3C—Mixed failure; and 3D—cohesive failure on dentin.

## 4. Discussion

Considering the studies of bond strength on resin/dentin interfaces, the quality of the dentin substrate can play a key role in the longevity of the bonding [24,27,28,29]. In the present study, we have evaluated the influence of artificial caries development models (chemical and biological) and substrate conditions on resin/dentin bond strength. In this study, the first null hypothesis that there is a difference between DCLL models producing was proved, since there was no significant influence of the DCLL model on μTBS of demineralized dentin. However, the second hypothesis that there is influence of DCLL model and demineralized dentin treatment on μTBS of an etch and rinse adhesive system was rejected, since the µTBS values were dependent of DCLL model and mineralizing agent type. The highest µTBS were found when demineralized dentin was treated with for Curodont™ Repair and NaF (*p* < 0.001), although there was no significant difference on µTBS when dentin was treated with MI Paste (*p* > 0.05).

This study corroborates previous investigations demonstrating that the bonding procedures on demineralized dentin [30,31,32] present lower µTBS when compared to a sound one, regardless of the artificial caries development model (Table 2). Morphological changes in the substrate provided by caries production process can induce a decreased µTBS [20,33]. This reduction can be associated with changes in physical and chemical properties of the demineralized substrate when compared to sound dentin [34]. Demineralized dentin provides a high porosity in the inter-tubular dentin, exposure of collagen fibers along with decrease in mineral content [35] and partial penetration of resin monomers and a non-homogeneous hybrid layer [36]. In a porous hybrid layer, over time, mineral and organic matrix would be degraded giving rise to gaps which may be visible using a SEM which show a higher rate of degradation [36]. In addition, the demineralization of dentin surface results in a more hydrophobic surface, avoiding the wettability of the adhesive [17].

The caries lesion provided by a biological model, which uses *S. mutans* biofilm, seems to be quite similar to the natural ones, based on molecular and structural evaluations [23]. Another model used for providing dentin caries-like lesions is the chemical one, and it can be used to simulate caries-affected dentin [5].

It is desirable that bonding between mineralized tooth tissues, such as dentin, and the restorative materials must be sufficiently effective to resist varied challenges, such as biofilm attack, hydrolytic and enzymatic degradation, thermal and mechanical stress from repeated loading over many months or years [37,38]. The reinforcement of demineralized collagen matrix can be achieved using remineralizing agents [10,39]. The current biomimetic remineralization approach provides a proof-of-concept that utilizes nanotechnological principles to mimic natural biomineralization, extending the longevity of resin–dentin bonds [10]. The mineral reinforcement of collagen matrix found in demineralized dentin appears to be a strategy that restores conditions found on sound dentin, as seen in the present study. In the present study the biomimetic remineralization strategy provided a higher (CPP-ACP + Chemical and Biological DCCL models; P_11_-4 + Biological DCCL) or similar (P_11_-4) μTBS to demineralized dentin than sound one, while the NaF remineralization provided higher μTBS values than demineralized dentin, but lower than sound one. It can be observed that only for CCP-ACP, regardless DCLL model, the mineralizing agent provided a higher μTBS than sound dentin.

Despite it, concerning affected dentin, Bahari et al. (2014) [40] showed that 5 consecutive days of CPP-ACP application for 15 min did not have any significant effect on μTBS of SB to demineralized dentin. However, it has been considered that the methodology used in that study was quite different from that used in the present one. Firstly, according to the methodology description (Bahari et al, 2014) even sound dentin was submitted to the CPP-ACP action. Casein phosphopeptides (CPP) have been described to bind amorphous calcium phosphate, forming nano-complexes of casein phosphopeptide–amorphous calcium phosphate (CPP-ACP), thereby stabilizing in calcium phosphates [41,42]. Calcium and phosphate ions can easily diffuse into the porous lesion and deposits in the partially demineralized crystals and rebuild hydroxy-apatite crystals [43]. This further substantiates the theory that CPP-ACP is considered a biomaterial [44]. The presence of bioavailable calcium and phosphate present the MI Paste™ can maintain a supersaturated state in dental substrate [11]. Studies have demonstrated that CPP-ACP could reduce demineralization and increase remineralization of dentin [44,45]. It is possible that in that study the CPP-ACP could have been impregnated onto caries-affected dentin and sound dentin [13,46] in the same way.

It is well-established that the collagen matrix serves as a scaffold for crystal deposition but does not provide a mechanism for orderly nucleation of hydroxyapatite [39]. The results of the present study can be attributed to the ability of CPP-ACP to increase deposition of crystals on the dentin surface [47]. Furthermore, the CPP also has the capacity to stabilize nano-ACP [48]. Therefore, the deposition and stabilization may result in a restructuring of the characteristics found in sound dentin, showing the highest µTBS when demineralized dentin was treated with MI Paste. This reinforcement approach of demineralized collagen matrix structure may favor the bonding procedure. Further studies should be carried out to verify the stability of the bonding strength when CPP-ACP is used.

It is generally believed that extracellular matrix proteins, which play an important role in controlling apatite nucleation and growth in the dentin remineralization process [49], mediate a biomineralization process. Biomimetic remineralization represents a different approach to this by attempting to backfill the demineralized dentin collagen with liquid-like ACP nanoprecursor particles that are stabilized by biomimetic analogs of noncollagenous proteins [10,50]. In this way, maybe this particular nucleation would provide a regular and feasible restructuration of the demineralized dentin and also would provide a favorable substrate for bonding, due to the more hydrophyllic nature of the substrate [23].

Another interesting finding of the present study was the fact that the µTBS means of MI Paste™ group was higher than those found in sound dentin, and did not show a significant difference between either artificial caries development models. The remineralization process and the artificial caries development model, studied in this article, showed that the artificial caries development model affects only the μTBS of the demineralized dentin treated with NaF and P_11_-4.

Similar to CCP-ACP, the P_11_-4 approach using the DCLL chemical model provided significantly higher μTBS than sound dentin. The chemical model of DCLL provided a lower content of type I collagen and higher content of calcium and phosphate ions, than the Biological one [23]. The collagen is the precursor for mineralization, acting as a scaffold for mineral aggregation. However, when NaF is used, only a deposition of ions and CaF formation occurs on dentin surface. Possibly the high content of mineral ions decreased the surface energy of the demineralized dentin [23]. The opposite can be observed when biomimetic remineralization happens, using CPP-ACP or P_11_-4. In this case, the mineralization occurs by organized crystal formation guided by the scaffold. This kind of surface can experience a high surface energy and a high level of wettability by resin monomers providing the highest µTBS [23].

The results of the study indicated that treatment with P_11_-4, with a single application showed significant improvement on µTBS. Despite the fact that there was significant difference between the DCLL models, when P_11_-4 is used, the µTBS values still show high values. For the biological model when the peptide P_11_-4 was used no significant difference from sound dentin was observed. It is suggested that the use of the P_11_-4 is able to nucleate hydroxyapatite and to promote repair of caries-like lesions in vitro. We have no knowledge in the literature of any report in which treatment with P_11_-4 has been conducted in demineralized dentin, associating its effects with bonding procedures. However, research groups using other peptides, observed that this strategy mimics the functions of non-collagenous proteins (NCPs) [51,52]. This suggests that the action of the P_11_-4 peptide reflects the reinforcing of demineralized collagen matrix.

The potential for enamel lesion repair of P_11_-4 may mimic the functions of NPCs [15,53,54]. Several studies indicate that P_11_-4 forms three dimensional fibrillar hierarchical structures resulting in gels in response to specific environmental triggers [53,54]. Assembled P_11_-4 forms scaffold-like structures with negative charge domains, mirroring biological macromolecules in mineralized tissue extracellular matrices (ECM) [54].

Fluoride is well-known for its proved anti-cariogenic and antimicrobial capacity. Its ability to prevent demineralization and promote remineralization by calcium phosphate precipitation on dental surface by reducing the dissolution of hydroxyapatite [55]. Thus, the effect of demineralized dentin treated with NaF on the µTBS, improved the µTBS compared to demineralized dentin, but did not reach the sound dentin µTBS. It has to be considered that the dentin etching with 35% phosphoric acid increases the bonding efficacy of dental adhesives and removes the smear layer and the superficial part of the dentin, opening dentin tubules, demineralizing the dentin surface and increasing the microporosity of the intertubular dentin [56]. Despite the phosphoric acid used in the etching procedure removing part of the mineral deposits, possibly the reinforcement provided by NaF on demineralized dentin structure would improve the µTBS compared to demineralized dentin [57,58]. However, the differences found with µTBS of Chemical and Biological models can be explained by the deeper demineralization provided by biological model than the chemical one. Therefore, it is known that the mineral deposition of fluorides occurs on surface and generally causes hyper mineralization of the dentin and in dentin tubules [10,59,60,61]. The disorganized precipitation and deposit of mineral on dentin may mechanically obliterate the tubules reducing the performance of the restorative material [61], providing less efficiency of NaF treatment.

Another important aspect is that the NaF and Curodont Repair treatments showed a statistically significant difference between the DCLL. With regard to the treatment with NaF, a previous study [62] showed that this treatment is able to reduce the subsurface dentin demineralization compared with the control from 30 to 50 µm depth. At the other depths (60–220 µm) NaF showed no positive effect. Such results may be attributed to a possible reaction between NaF and demineralized dentin, producing soluble fluoride. In the present study, the demineralized dentin associated with NaF treatment exhibit mixed failure type. This result may be associated with a surface reinforcement, which was partially removed by the etching with the phosphoric acid during bonding procedures.

With the exception of the treatment with NaF, demineralized dentin treated with Curodont Repair has shown no negative influence on bond strength, as the biological DCLL did not differ from sound dentin. This behavior can be attributed to different interactions with the demineralized substrate, since the caries depth produced by the biological model DCLL was higher than chemical one. It has been reported that the P_11_-4 scaffold can act as nucleator for hydroxyapatite, infiltrating into the porous lesions and increase the mineral diffusion within the lesion, restructuring the affected tissue [15].

Moreover, regardless of the artificial caries development model, the results presented in this study suggest that the use of remineralizing agents can reinforce the mechanical properties of demineralized dentin and would favor the durability of resin-dentin bonds, since the treated demineralized substrate provides an organized mineral surface. However, the degree of improvement in bonding strength is dependent on the artificial caries development model and dentin treatment. Further studies have to be carried out in order to observe the long-term efficacy of the remineralized dentin bonded to adhesive systems.

## 5. Conclusions

Based on the results of this study it can be concluded that:

MI Paste™ and Curodont™ Repair recovered higher μTBS values when compared to sound dentin. Each agent shows a different interaction with each artificial caries development model used. The remineralizing treatment of demineralized dentin is a potential approach for increasing bond strength of etch and rinse adhesive systems.

## Figures and Tables

**Table 1:** Materials, manufactures, components, batch numbers and application mode of tested materials.

Materials (Manufactures)	Main Components	Batch Number	Application Mode
**0.2% NaF Solution**	0.2 g of NaF in 100 mL deionized water	Made in the Lab *	1. Apply 1.0 mL of 0.2% NaF solution
**Ca^2+^ and PO_4_^3−^ Solution**	Saturated solution of Ca^2+^ and PO_4_^3−^ (1.5 mmol/L calcium, 0.9 mmol/L phosphate, and 150 mmol/L KCl in 20 mmol/L cacodylic buffer, pH 7.0) [24].	Made in the Lab	1. Apply 0.1 mL of Ca^2+^ and PO_4_^3−^ solution
**MI™ Paste**—GC Internacional, Itabashi-ku, Tóquio, Japão	Glycerol, CPP-ACP, D-Sorbitol, Propylene glycol, Titanium dioxide and silicon	N2347319	1. Apply 0.1 mL of MI™ Paste
**Curodont™ Repair**—Credentis AG, Dorfstrasse, Windisch, Switzerland	P_11-_4 peptide—amino acid sequence—(Ace-Gln-Gln-Arg-Phe-Glu-Trp-Glu-Phe-Glu-Gln-Gln-NH_2_)	N342x	1. Apply 50 µL of Curodont™ Repair for 5 min 2. Apply 0.1 mL of Ca^2+^ and PO_4_^3−^ solution
**Scotchbond™ Universal Etchant**—3M ESPE; St Paul, MN, USA	32% phosphoric acid	N345	1. Apply etchant for 15 s 2. Rinse for 10 s
**Adper Single Bond 2.0**—3M ESPE; St Paul, MN, USA	HEMA, water, ethanol, Bis-GMA, dimethacrylates, amines, metacrylate functional copolymer of polyacrylic and polyitaconic acids, 10% by weight of 5 nanometer-diameter spherical sílica particles	N42912	3. Blot water excess 4. Apply 2 consecutive coats of adhesive for 15 s with gentle agitation 5. Gently air dry for 5 s 6. Light-cure for 10 s
**Filtek™ Z350 XT**—3M ESPE; St Paul, MN, USA	BIS-GMA, Bis-EMA, UDMA, TEG-DMA, camphorquinone, non-agglomerated silica nanoparticles	N98354	1. Incremental insertion 2 mm 2. Light-cure for 20 s

* Pediatric Dentistry Laboratory.

**Table 2:** Average and standard deviation of μTBS of demineralized dentin considering the Artificial Caries Development Models.

Experimental Groups	Artificial Caries Development Models	
	Chemical Model	Biological Model
**Sound Dentin**	43.32 ± 4.35	
**Demineralized Dentin**	21.96 ± 5.92 Ca *	22.89 ± 2.68 Da *
**Demineralized Dentin + NaF**	33.43 ± 10.42 Ba *	26.94 ± 6.70 Cb *
**Demineralized Dentin + MI Paste™ (CPP-ACP)**	45.25 ± 8.83 Aa *	47.95 ± 6.69 Aa *
**Demineralized Dentin + Curodont™ Repair (P_11_-4)**	46.42 ± 12.03 Aa *	42.07 ± 7.83 **Bb**

Uppercase letters represent statistically significant difference in the column (*p* < 0.001). Lowercase letters represent no statistically significant difference in the row (*p* > 0.05). * indicates statistically significant difference with the control group (sound dentin) (*p* < 0.05) by additional Dunnett’s test.
